# Supplementary material for: Diurnal rhythms of choice: a novel state-dependent drift diffusion model uncovers time-dependent changes in rat decision making
Source: Res Sq. 2026 Jun 2:rs.3.rs-9883645. Preprint. [Version 1] doi: 10.21203/rs.3.rs-9883645/v1 (PMC13252520; doi:10.21203/rs.3.rs-9883645/v1)
Supplement: 1 [file NIHPPRS9883645V1-supplement-1.pdf]

Appendix A Supplementary Figures

A

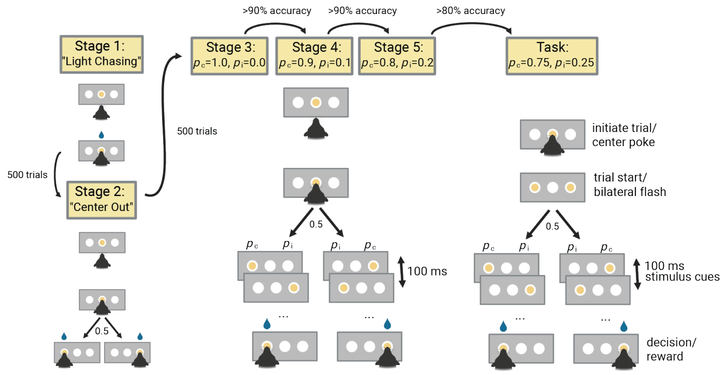

B

Rat ID	*n* Trials	*n* Days
1062	94,387	86
1052	68,394	88
1064	66,524	88
1060	56,849	86
1065	45,974	58
1072	45,003	70
1054	40,919	84
1029	27,385	85
Remy	23,482	10
Rudy	20,915	10
Robert	18,667	10
Rhea	17,213	10
1059	17,168	66
Dopey	12,774	10
Draco	12,453	10
Ron	10,679	8
Randy	8,382	7
Denna	5,093	10

Fig. S1 Animal training procedure and dataset summary. (A) Animals are trained in a six-stage procedure. In stage 1 (light chasing), animals nose poke the illuminated center port to receive a reward (500 trials). In stage 2 (center-out), animals nose poke the center port and then either the left or right port (assigned randomly; 500 trials). In stages 3–5, animals initiate trials via the center port and must achieve >90% accuracy over 500 trials by selecting the port with the higher flash probability. Before advancing to the performance stage (stage 6), animals must achieve >80% accuracy over 500 trials. **(B)** Summary statistics for the 18 rats included in the study.

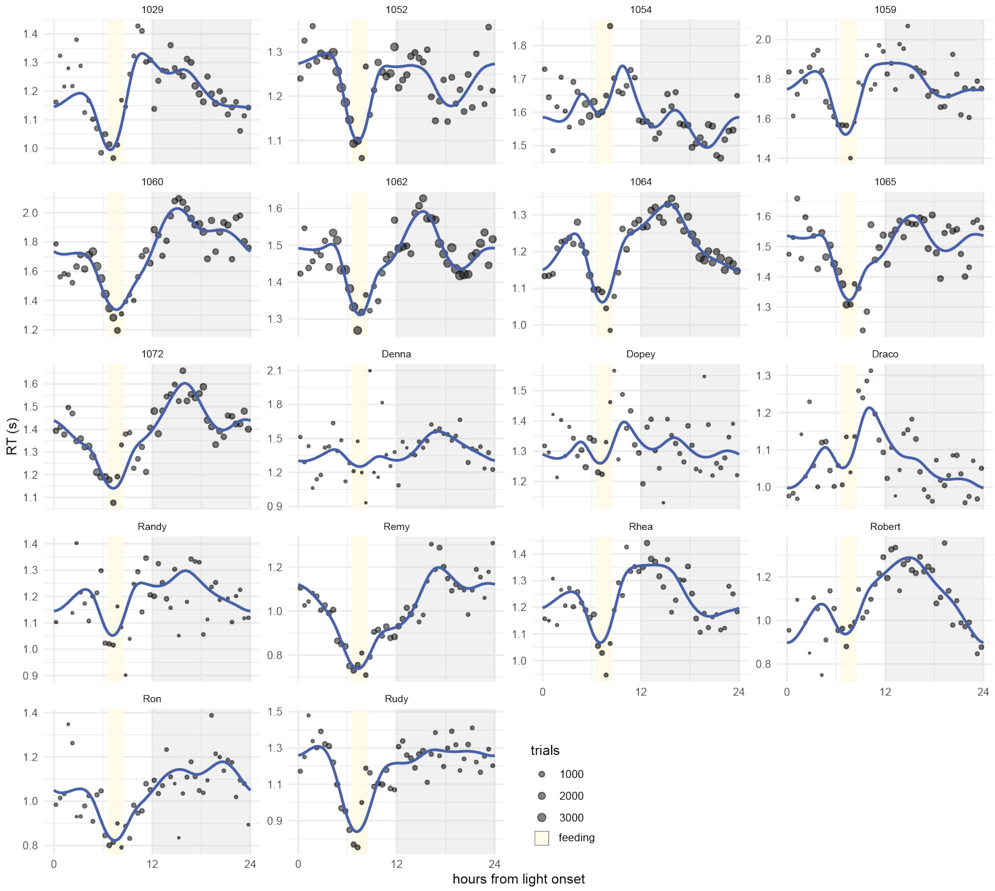

Fig. S2 Individual GAM fits of reaction times across the 24-hour cycle. Blue lines indicate animal-level averages; points are scaled by the number of trials contributing to each estimate; yellow bars denote feeding periods.

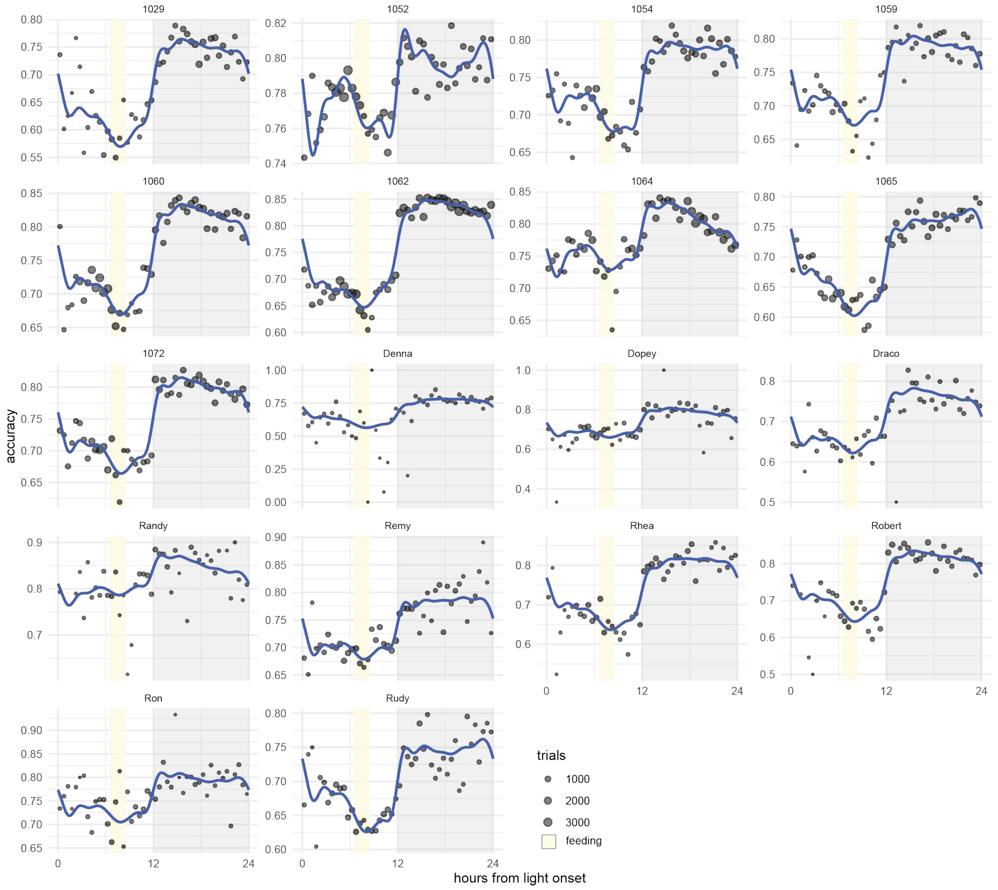

Fig. S3 Individual GAM fits of accuracy across the 24-hour cycle. Blue lines indicate animal-level averages; points are scaled by the number of trials; yellow bars denote feeding periods.

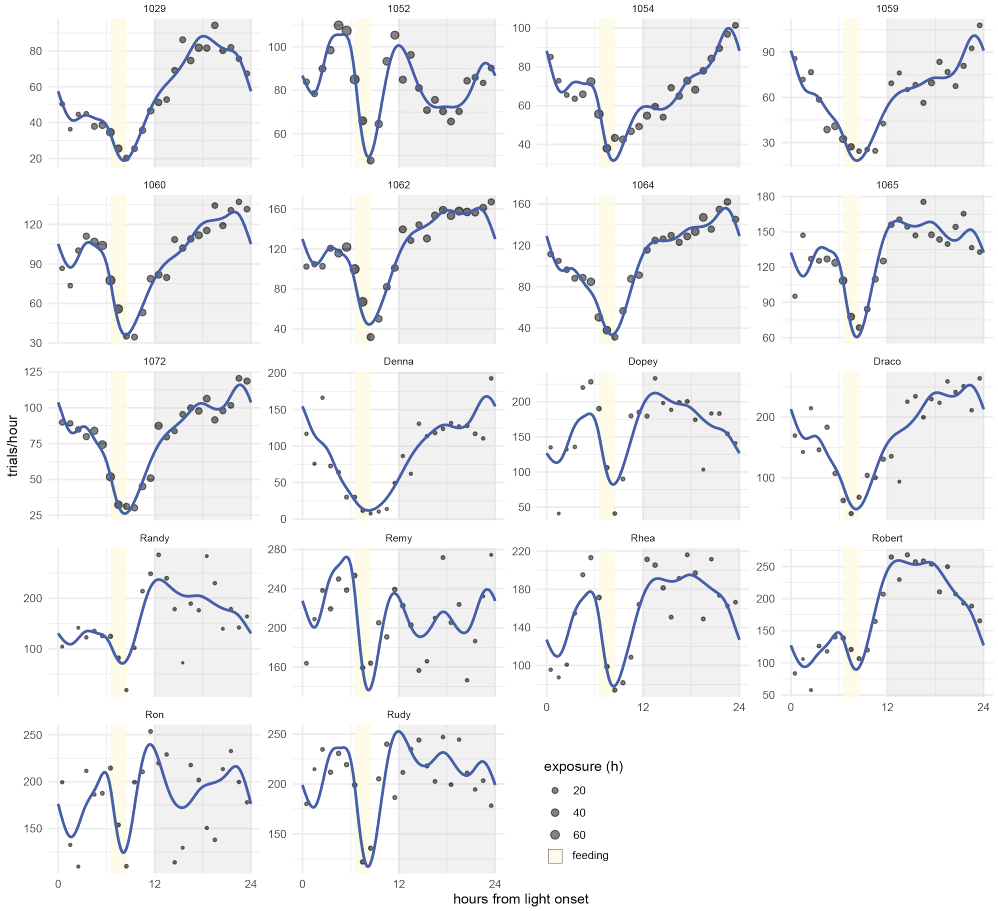

Fig. S4 Individual GAM fits of trial production across the 24-hour cycle. Blue lines indicate animal-level averages; points are scaled by the number of trials; yellow bars denote feeding periods.

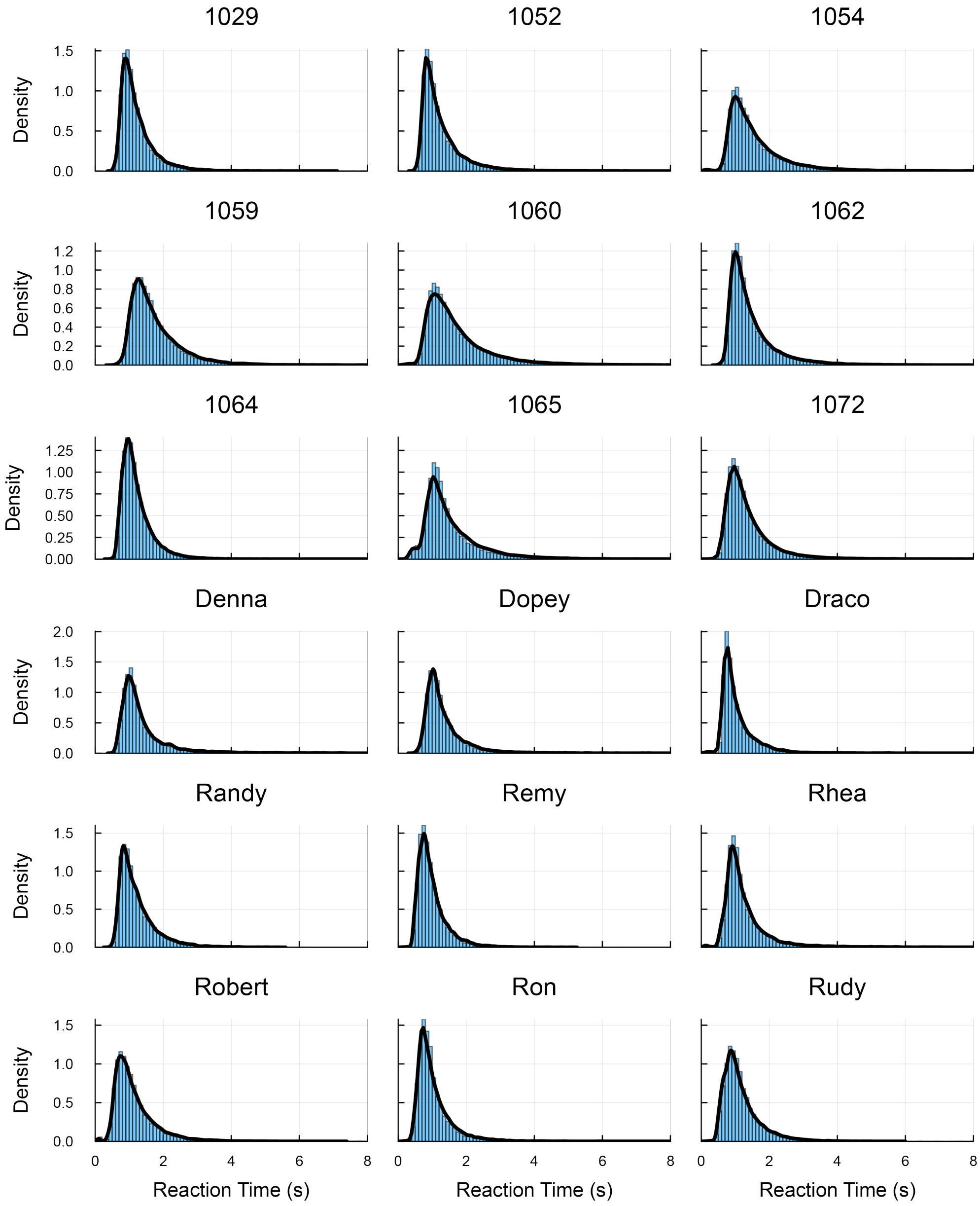

Fig. S5 The DDM-HMM captures RT distributions. Simulations from learned DDM-HMMs (black line) closely match the empirical data (blue histogram) from all animals.

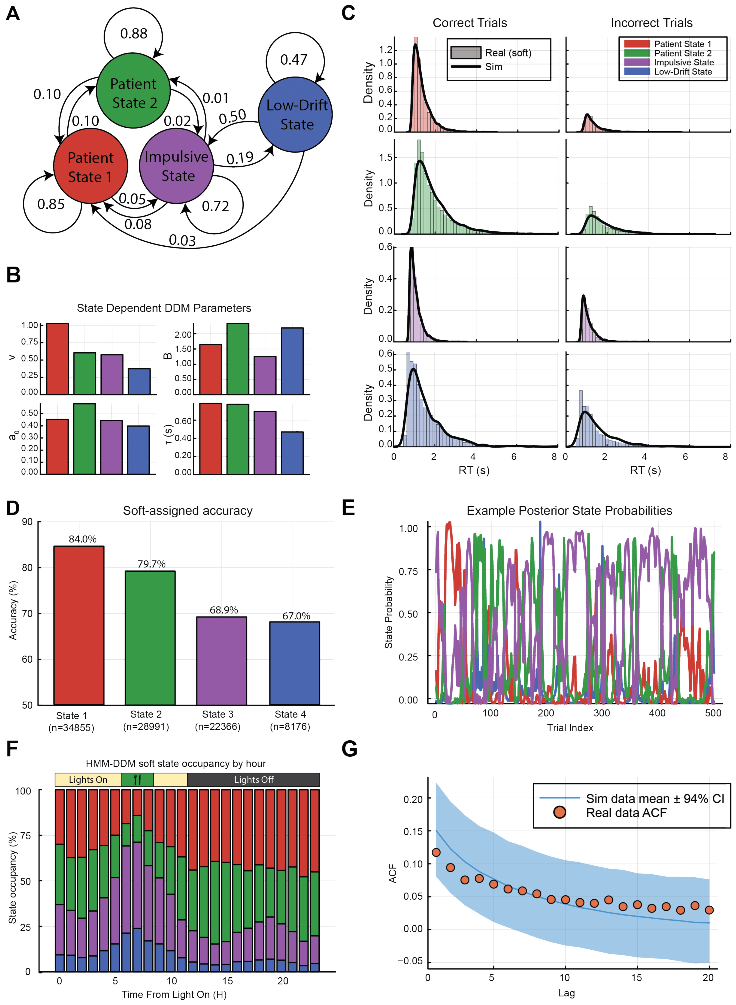

Fig. S6 Example DDM-HMM fit for an expert rat. **(A)** Schematic of the transition dynamics between the four inferred latent decision states: patient state 1, patient state 2, impulsive state, and low-drift. **(B)** State-dependent DDM parameters (drift rate, boundary separation, starting bias, and non-decision time). **(C)** RT distributions for correct/incorrect trials, shown for each state. Histograms reflect real data using soft state assignments; black curves indicate simulated data from the fitted HMM–DDM. **(D)** Soft-assigned choice accuracy for each state. **(E)** Example posterior state probabilities across trials. **(F)** Hourly state occupancy relative to the light–dark cycle. **(G)** ACF of inferred states in the real data (points) compared with simulated data from the model (mean ± 94% CI).

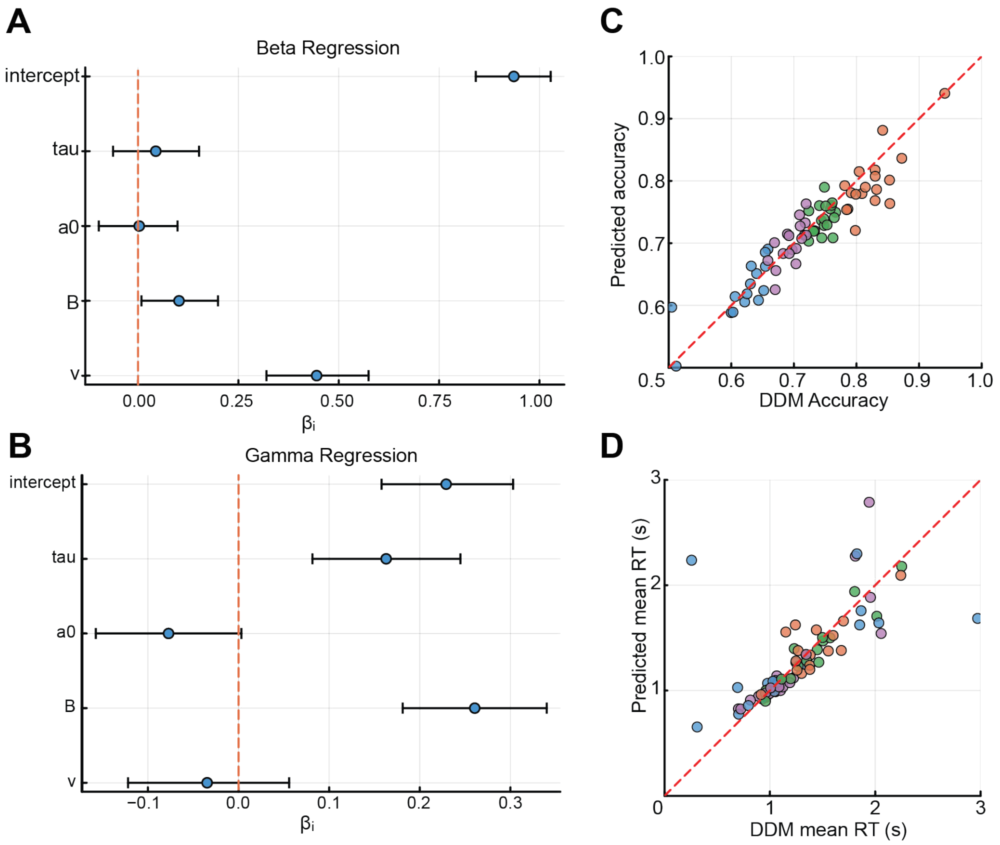

Fig. S7 DDM parameter regressions. (A) Forest plot of posterior mean coefficients and 95% credible intervals for beta regression predicting accuracy from DDM parameters. **(B)** Forest plot predicting mean reaction time from DDM parameters. **(C)** Observed versus predicted accuracy from the beta regression. **(D)** Observed versus predicted reaction time from the gamma regression.

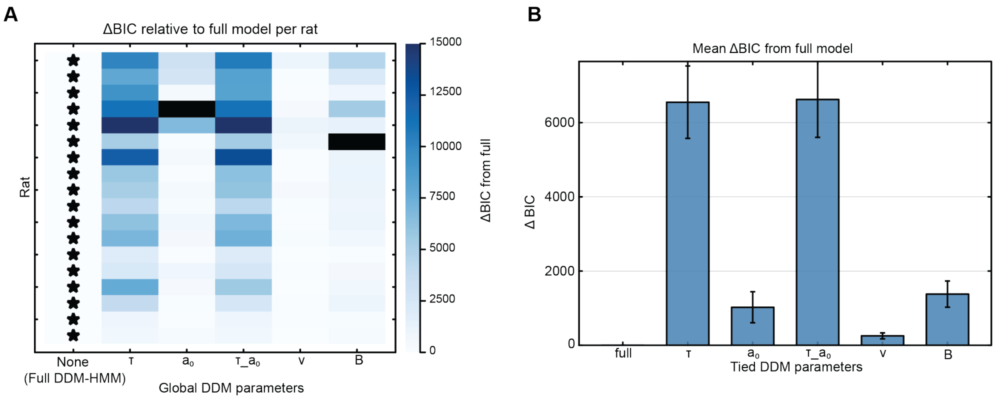

Fig. S8 BIC comparison suggests the full DDM-HMM is the best model of animal 24-hour decision making data. (A) Heatmap of individual animal ΔBIC from the "full" model. Black stars indicate the best fitting model. Bluer colors represent worse model fits compared to the full model. **(B)** Mean ΔBIC at the population level.

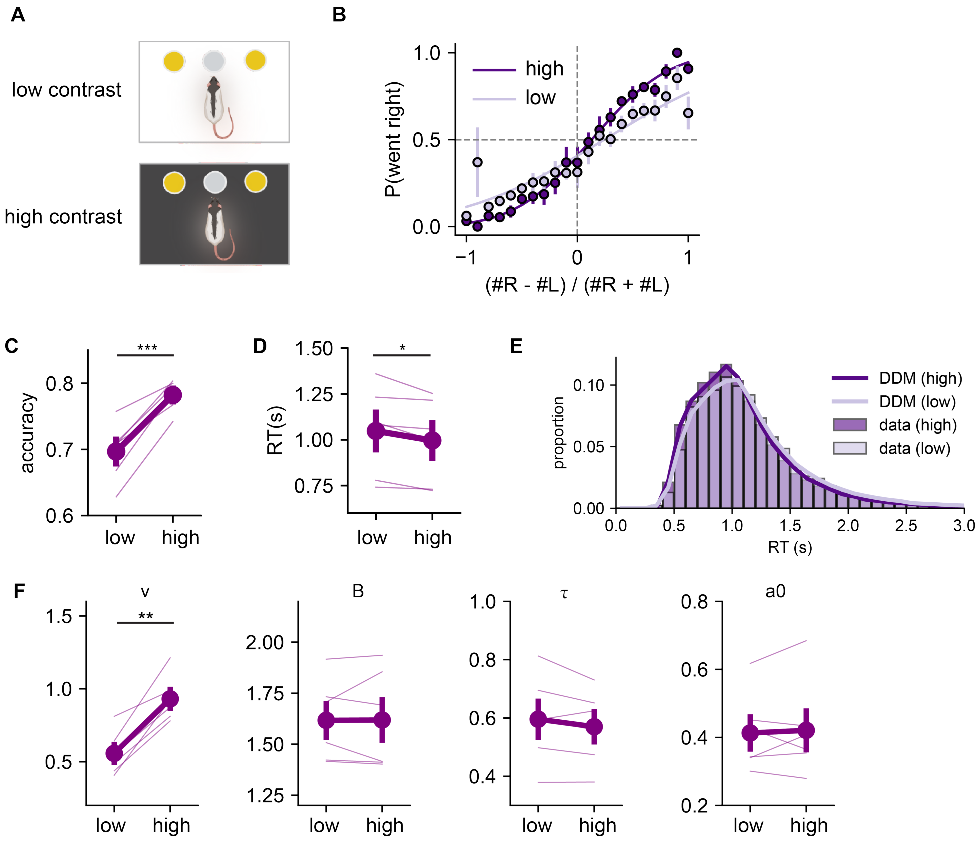

Fig. S9 Rats show a higher drift rate under high contrast conditions during daily 2-hr training. **(A)** Schematic of rats training under low contrast (room light on) and high contrast (room light off). **(B)** Psychometric curves under low and high contrast conditions. **(C)** Average accuracy across animals ($\pm$ SE; $n = 6$). **(D)** Average RT across animals ($\pm$ SE; $n = 6$). **(E)** RT distributions from low and high contrast. Bars represent the data, lines represent DDM predictions. **(F)** Mean fitted DDM parameters for drift rate (v), boundary separation (B), non-decision time (τ) and starting point (a_0).

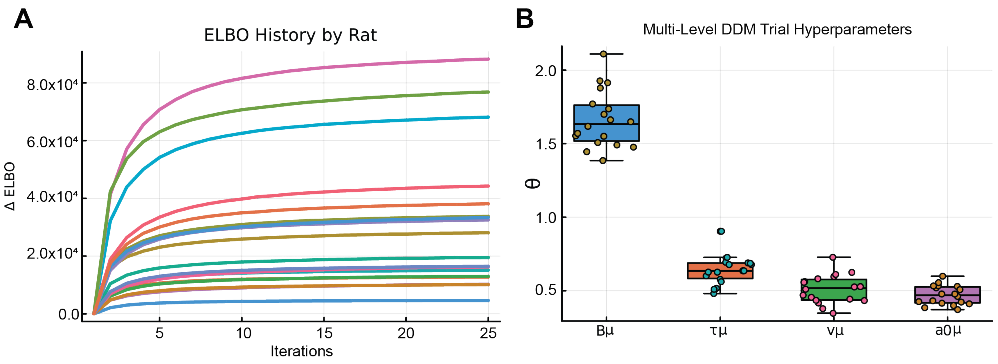

Fig. S10 Parameter learning in a multi-level DDM model. **(A)** Change in evidence lower bound (ΔELBO) across optimization iterations for each rat. **(B)** Posterior distributions of trial-level hyperparameters from the multilevel DDM.

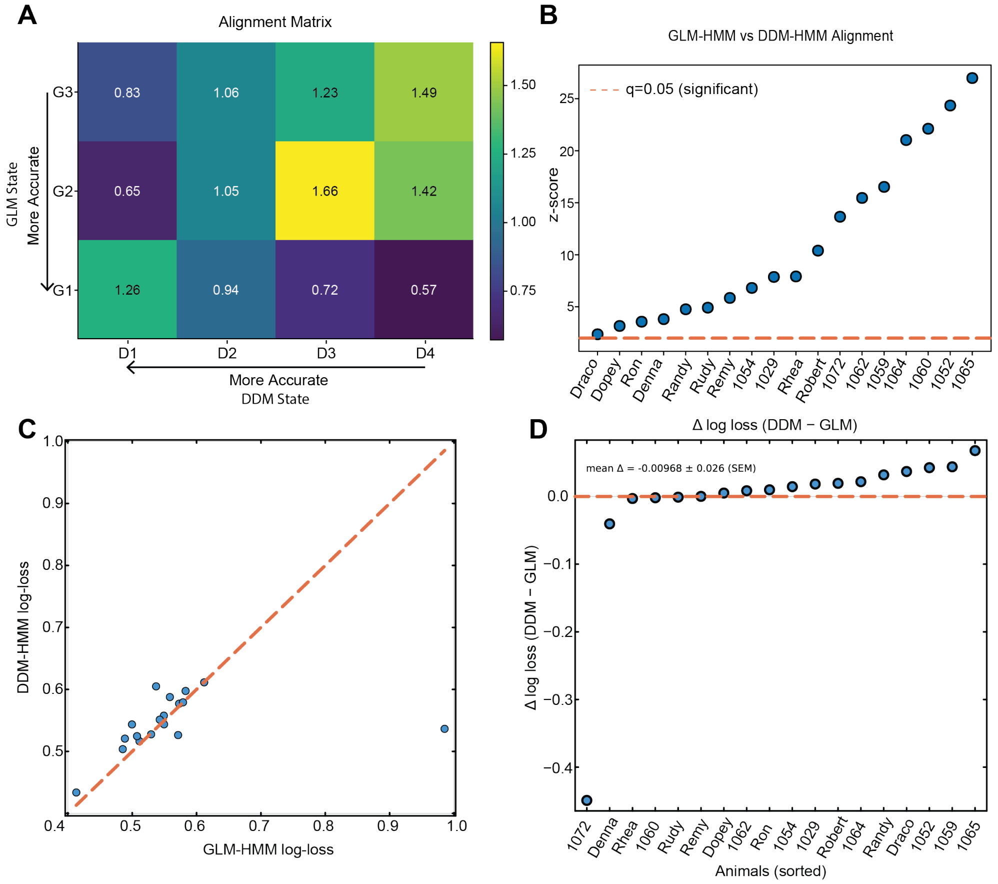

Fig. S11 The DDM-HMM and GLM-HMM find similar latent dynamics. **(A)** Alignment matrix showing above-chance joint state activity. **(B)** Permutation test results for NMI between the GLM-HMM and DDM-HMM posteriors. **(C)** Model choice prediction performance comparison. **(D)** Difference in log-loss between models.

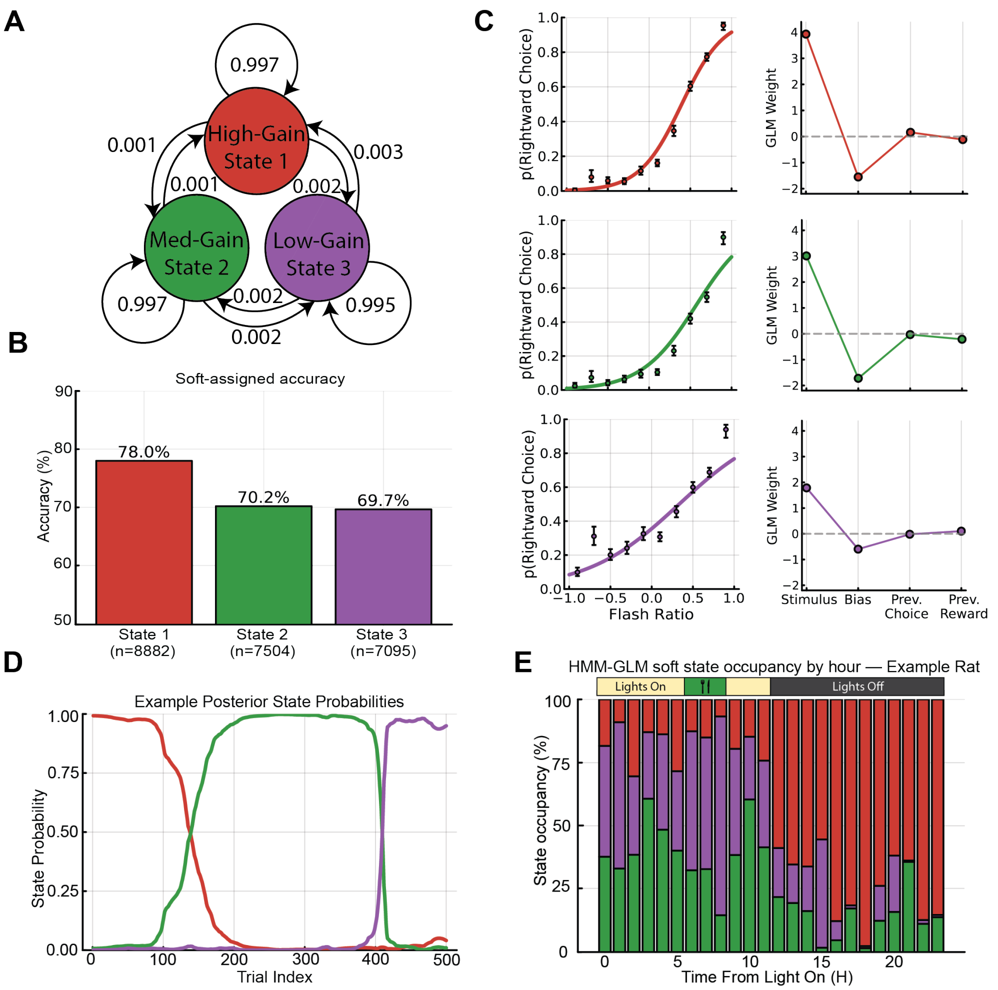

Fig. S12 GLM-HMM latent states align with task structure. **(A)** State transition diagram for a three-state GLM-HMM. **(B)** Soft-assigned choice accuracy for each GLM-HMM state. **(C)** Psychometric functions (left) and GLM weights (right) for each state. **(D)** Example posterior state probabilities across trials. **(E)** State occupancy as a function of time from lights on.

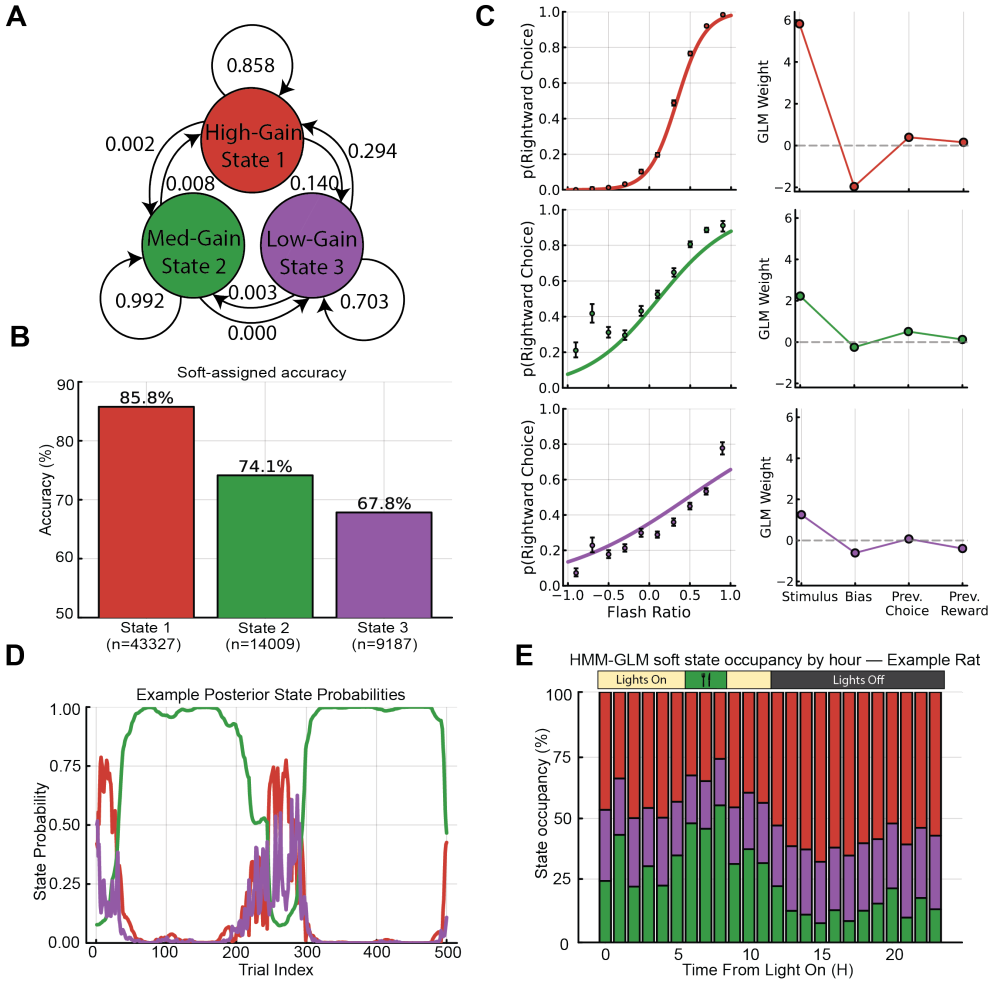

Fig. S13 Replication of GLM-HMM state structure in an expert rat. **(A)** State transition structure for a three-state GLM-HMM. **(B)** Soft-assigned accuracy across states. **(C)** State-specific psychometric functions and GLM weights. **(D)** Posterior state probabilities across trials. **(E)** Circadian modulation of GLM-HMM state occupancy.
